# Association between Low Dietary Protein Intake and Geriatric Nutrition Risk Index in Patients with Chronic Kidney Disease: A Retrospective Single-Center Cohort Study

**DOI:** 10.3390/nu8100662

**Published:** 2016-10-23

**Authors:** Aki Kiuchi, Yasushi Ohashi, Reibin Tai, Toshiyuki Aoki, Sonoo Mizuiri, Toyoko Ogura, Atsushi Aikawa, Ken Sakai

**Affiliations:** 1 Department of Nephrology, Omori Medical Center, School of Medicine, Toho University, Tokyo 143-8541, Japan; aki.futaki@med.toho-u.ac.jp (A.K.); reibin_1027@med.toho-u.ac.jp (R.T.); toshi.a.77@med.toho-u.ac.jp (T.A.); aaikawa@med.toho-u.ac.jp (A.A.); kensakai@med.toho-u.ac.jp (K.S.); 2 Department of Nephrology, Sakura Medical Center, School of Medicine, Toho University, 564-1, Shimoshizu, Sakura, Chiba 285-8741, Japan; 3 Division of Nephrology, Ichiyokai Harada Hospital, Hiroshima 731-5134, Japan; sm210@med.toho-u.ac.jp; 4 Department of Nutrition, Omori Medical Center, Toho University, Tokyo 143-8541, Japan; ogura-tyk-000@med.toho-u.ac.jp

**Keywords:** body mass index, body composition, chronic kidney disease, food intake, geriatric nutritional risk index, nutritional status, serum albumin, wasting syndrome

## Abstract

Reduced dietary protein intake in malnourished patients with chronic kidney disease (CKD) may be associated with adverse clinical outcomes, which may mask any efficacy of a low-protein diet. The study included 126 patients with CKD who attended a dedicated dietary counseling clinic in 2005–2009 and were systematically followed until January 2015. Of these patients, 20 (15.9%) had moderate or severe nutrition-related risk of geriatric nutritional risk index (GNRI) < 92; these patients were more likely to be older, have a greater proteinuria, and have lower body mass index and serum albumin concentration. Dietary protein intake was significantly lower in older patients (*r* = −0.33, *p* < 0.001) and those with lower glomerular filtration rate (*r* = 0.47, *p* < 0.001). The non-protein to nitrogen calorie ratio was independently associated with GNRI. Reduced GNRI was significantly associated with mortality (hazard ratio (HR) = 4.94; 95% confidence interval (CI) = 1.61–15.42, *p* = 0.012) and cardiovascular events (HR = 9.37; 95% CI = 2.49–37.34, *p* = 0.006), but not with adverse renal outcomes. Restricting protein intake may be harmful to patients with any nutrition-related risk, suggesting that improvement of nutritional status should be a high priority.

## 1. Introduction

Clinical practice guidelines for adult patients with chronic kidney disease (CKD) have recommended dietary protein intake of 0.6–0.8 g/kg body weight per day and energy intake of 30–35 kcal/kg ideal body weight per day [1,2]. However, protein restriction is only a part, though a very relevant part, of a more complex dietary management of CKD patients [3,4,5]. Phosphate intake should be reduced (700–400 mg/day), as well as sodium intake (2–3 g/day). Dietary energy intake must cover energy requirements up to 35 kcal/kg/day for patients <65 years and 30 kcal/kg/day for patients >65 years old. Qualitative aspects of foods (essential amino acid and ketoacids, calcium carbonate, vitamins, iron) are also important. These findings indicated the importance of maintaining adequate energy intake in patients on restricted dietary protein, by increasing caloric intake from carbohydrates and/or fats.

To date, many prospective cohort studies have evaluated the association between body mass index (BMI) and mortality in the Japanese population [6,7,8,9,10,11]; some showed a U-shaped or reverse J-shaped association. The lowest risk of total mortality and mortality was observed for a BMI of 21 to 27 kg/m^2^ in middle-aged and elderly Japanese [12]. In overview of dietary reference intakes for Japanese 2015, the target BMI was recommended as 18.5–24.9 kg/m^2^ in the age of ≤49, 20.0–24.9 kg/m^2^ in the age of 50–69, and 21.0–24.9 kg/m^2^ in the age of ≥70, considering the necessity to take into account both the prevention of frailty and prevention of life-style related diseases [13]. Protein-energy wasting (PEW) is common in patients with CKD and is associated with an increased risk of death [14]. Dietary protein and energy intake may be inadequate in patients with advanced CKD due to uremic anorexia, inter-current illness, systemic inflammation, and/or dietary protein restriction. A consensus statement by the International Society of Renal Nutrition and Metabolism described the advantages of oral nutritional supplements, including their efficacy, safety, and compliance. Anabolic strategies, alone or combined with nutritional supplementation, have been shown to improve protein stores and PEW [14]. However, it is difficult to replenish protein and energy stores in CKD patients, especially in older patients, on a restricted protein diet. Reduced dietary protein intake by malnourished patients may also be associated with adverse clinical outcomes, which may mask the efficacy of low-protein diets.

A multidisciplinary approach is required to reliably evaluate nutritional status. The geriatric nutritional risk index (GNRI), consisting of BMI and serum albumin concentration, is a simple tool that assesses nutrition-related risk and may indicate reduced body stores of protein and energy [15,16,17]. The ability of the GNRI to predict the risk of mortality in patients with CKD, including those with massive proteinuria, has not yet been determined.

The goals of the present study were: (1) to evaluate the associations among aging, kidney function, and dietary intake in patients with CKD; (2) to determine the associations of GNRI with body composition, mortality, cardiovascular events, and adverse renal outcomes in these patients; and (3) to determine the association between dietary intake and GNRI in patients with CKD.

## 2. Materials and Methods

### 2.1. Study Design

This study was approved by the Ethics Committee of Toho University Omori Medical Center, Tokyo, Japan (approval number: 25-252) and adhered to the principles of the Declaration of Helsinki. Informed consent was obtained from all study participants.

Between 2005 and 2009, 175 patients with CKD aged ≥20 years attended a dedicated dietary counseling clinic, consisting of a nephrologist and two registered dietitians in our department. At the clinic, patients were advised to consume the recommended 0.6–0.8 g protein per kg ideal body weight per day and a total of 30–35 kcal total energy per kg ideal body weight per day. Patients were followed-up approximately once every 1–2 months. Daily nutrient intake, estimated from 24-h dietary recall, and analysis using standard food composition tables was assessed in 126 of these patients (a median age (10th–90th percentile) of 67 years old (37–81 years old)). The median period between the first visit to the dietary clinic and the day of assessment of their dietary records was 1552 days (10th–90th percentile, 256–3330 days). A retrospective review of their medical charts identified 126 patients with complete clinical data, including anthropometric measurements, blood pressure, proteinuria, kidney function, and dietary intake of sodium, protein, and energy at the time of dietary nutrient assessment. Patients have been monitored until death, loss to follow-up, or January 2015, for a median 64 months (10th–90th percentile, 14–96 months).

Patient characteristics were recorded, including age, sex, height, body weight, BMI, body composition, underlying renal disease, and blood pressure. Serum concentrations of albumin, calcium, phosphorus, total cholesterol, triglycerides, fasting blood glucose, uric acid, C-reactive protein and creatinine (Cr) were measured, as were hemoglobin levels, estimated glomerular filtration rate (eGFR), urinary protein to creatinine ratio (UPCR) in random urine samples, and urea nitrogen appearance (UNA) estimated from 24 h urine collection tests. Age-specific underweight was defined as <18.5 kg/m^2^ in the age of ≤49, <20.0 kg/m^2^ in the age of 50–69, or <21.5 kg/m^2^ in the age of ≥70 for three age categories of adults, according to comprehensive investigation of BMI ranges with the lowest all-cause mortality reported in epidemiological observational studies and actual BMI of Japanese people [12]. Resistant hypertension was defined as uncontrolled blood pressure (office systolic blood pressure ≥130 mmHg or office diastolic blood pressure ≥80 mmHg) despite antihypertensive therapy using three or more medications, including diuretics. Controlled blood pressure using four or more drugs was also considered resistant hypertension [18,19]. Hyperuricemia was defined as uric acid concentrations >7.0 mg/dL in men and >5.7 mg/dL in women [20]. eGFR was calculated according to the revised formula for Japanese patients, as 194 × creatinine − 1.094 × age − 0.287 (×0.739 for women), according to the Modification of Diet in Renal Disease (MDRD) method [21]. Basal energy expenditure (BEE) was calculated using the Harris–Benedict equation as (66.47 + 13.75 × body weight (kg) + 5.0 × height (cm) − 6.76 × age (years)) in men and (655.1 + 9.56 × body weight (kg) + 1.85 × height (cm) − 4.68 × age (years)) in women [22]. GNRI was calculated as (14.89 + albumin (g/dL)) + (41.7 × body weight/ideal body weight). The ideal body weight was calculated using height and an idealized BMI of 22 kg/m^2^ [15,17]. Clinical characteristics and adverse clinical outcomes were compared in groups with GNRI < 92 (defined as moderate or severe nutrition-related risk) and ≥92 (defined as low or no nutrition-related risk) [23,24,25].

### 2.2. Assessment of Body Composition

Bioimpedance analysis (BIA) was performed at the time of dietary nutrient assessment in a standard manner with the patient lying supine on a flat, nonconductive bed for at least 15 min. A segmental BIA instrument (Inbody S20^®^; Biospace Co., Ltd., Seoul, Korea) with eight tactile electrodes used. The microprocessor-controlled switches and BIA analyzer were activated, and segmental resistances of the arms, trunk, and legs were measured at four frequencies (5, 50, 250, and 500 kHz), for a total of 20 segmental resistances per patient. Using the BIA software, the sum of the segmental resistances for each body segment was used to calculate total body water (TBW), intracellular water (ICW), and extracellular water (ECW) [26]. Body composition was separated into three components: (a) water-free mass, consisting of proteins, fats, and minerals; (b) ICW content; and (c) ECW content. Each measured fluid compartment was expressed as both the actual value and the percentage of body weight. The phase angle was calculated by using the sum of impedance and reactance of the right arm, trunk, and right leg and based on the following equation: Phage angle (°) = (Reactance/Resistance) × (180°/Π) [27].

### 2.3. Endpoints

The endpoint of the study was the time to the first recorded adverse event. Cox proportional hazard models were used to compare adverse renal outcomes, cardiovascular events, and all-cause mortality. Adverse renal outcomes were defined as a ≥50% decline in eGFR relative to baseline, initiation of dialysis therapy or renal transplantation [28,29]. Cardiovascular events were defined as a composite of hospital-treated myocardial infarction or coronary intervention, hospital-treated heart failure, and hospital-treated stroke.

### 2.4. Statistical Analyses

The measured values were expressed as the means ± standard deviations and percentages. Continuous variables in the low and high GNRI groups were compared using a linear regression model, whereas categorical variables were compared using Pearson’s chi-squared tests. Correlations between variables were determined using the Pearson product-moment correlation coefficient. Logistic and linear regression analyses were used to identify associations between a GNRI < 92 and demographic factors. Explanatory variables that showed significant correlations (*p* < 0.10) with a GNRI < 92 were entered into a multivariate analysis to evaluate independent associations. The Kaplan–Meier method was used to evaluate survival outcomes. Nutrition-related risk of a GNRI < 92 was tested in the Cox model to identify the association of the investigated outcomes, with the results expressed as hazard ratios (HRs) and 95% confidence intervals (CIs). A graphical approach (log–log plots) was used to assess the proportionality of HRs. Continuous variables were divided into two groups by the median value. All variables in HR were estimated satisfied this assumption. A probability (*p*) value < 0.05 was considered statistically significant. All statistical analyses were performed using JMP 12.0 statistical software (SAS Institute, Inc., Cary, NC, USA).

## 3. Results

### 3.1. Patient Characteristics at the Time of Dietary Nutrient Assessment

**Figure 1:**
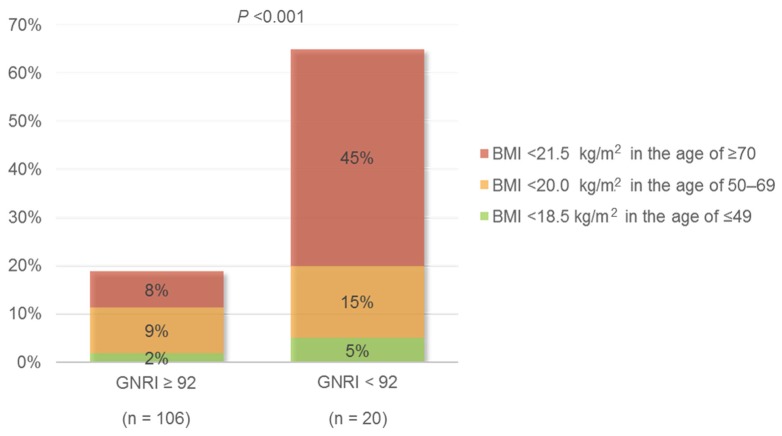
Age-specific underweight in patients with a GNRI ≥ 92 and < 92. Abbreviations: GNRI, geriatric nutritional risk index; BMI, body mass index.

**Figure 2:**
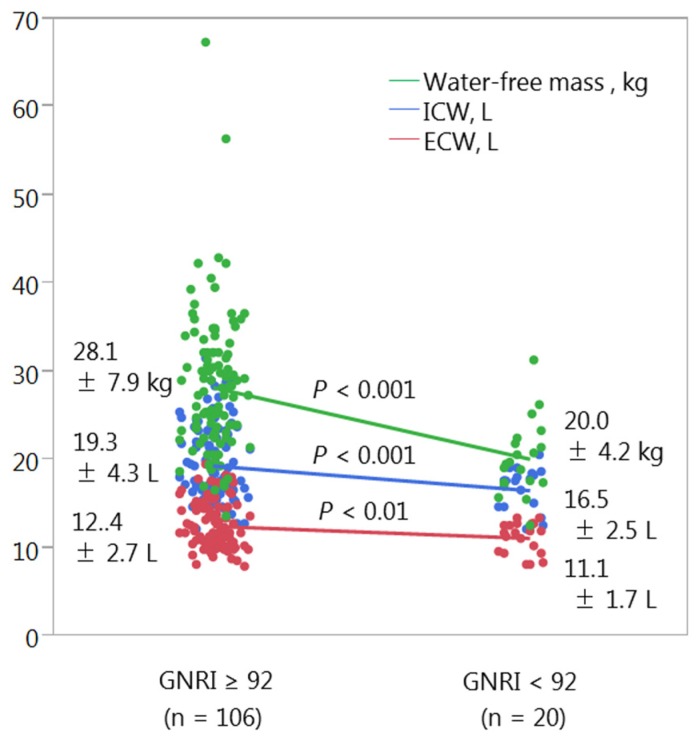
Body composition in patients with a GNRI ≥ 92 and < 92. Abbreviations: GNRI, geriatric nutritional risk index; ICW, intracellular water; ECW, extracellular water.

Table 1 shows the characteristics of the study population at the time of dietary nutrient assessment. Of the 126 patients, 20 (15.9%) had a GNRI < 92 and 106 (84.1%) had a GNRI ≥ 92. Patients with low GNRI were significantly older (69.7 ± 14.3 vs. 62.1 ± 16.3 years, *p* = 0.043) and had lower hemoglobin concentrations, and greater proteinuria (1.8 ± 2.1 vs. 0.8 ± 1.3 g/g Cr, *p* = 0.045) than patients with high GNRI. Patients in the low GNRI group also had significantly lower BMI (19.6 ± 2.1 vs. 23.3 ± 3.8 kg/m^2^, *p* < 0.001) and serum albumin concentrations (3.3 ± 0.4 vs. 4.0 ± 0.4 kg/m^2^, *p* < 0.001). Age-specific underweight, especially BMI < 21.5 kg/m^2^ in the age of ≥70, was more likely to be observed in the low GNRI group (65% vs. 19%, *p* < 0.001) (Figure 1). Assessments of body composition in the two groups showed that phase angle (5.1° ± 0.9° vs. 4.4° ± 0.6°, *p* < 0.001) and all three components, water-free mass (20.0 ± 4.2 vs. 28.1 ± 7.9 kg, *p* < 0.001), ICW content (16.5 ± 2.5 vs. 19.3 ± 4.3 L, *p* < 0.001), and ECW content (11.1 ± 1.7 vs. 12.4 ± 2.7 L, *p* < 0.01), were significantly lower in the low, than in the high, GNRI group (Supplementary Materials Table S1; Figure 2).

Patients with both low protein intake and inadequate calorie intake tended to be older and have lower GFR levels than patients with higher protein intake (Supplementary Materials Table S2).

### 3.2. Dietary Intake of Sodium, Protein, and Energy at the Time of Dietary Nutrient Assessment

Table 2 shows the dietary intake of sodium, protein, and energy in patients with a GNRI < 92 and ≥ 92. Both protein intake (41 ± 15 vs. 47 ± 15 g, *p* = 0.09) and calorie intake (1763 ± 129 vs. 1819 ± 189, *p* = 0.11) tended to be lower in the low, than in the high, GNRI group, but the differences were not statistically significant. The average non-protein calorie to nitrogen (NPC/N) ratio was significantly lower in the low GNRI group than in the high GNRI group (*p* < 0.05), due to the greater reduction in protein intake in patients in the low GNRI group. In both groups, however, the average calorie intake was 35 kcal/standard weight/day lower than the target level.

### 3.3. Associations of Protein and Calorie Intake with Age and Kidney Function

**Figure 3:**
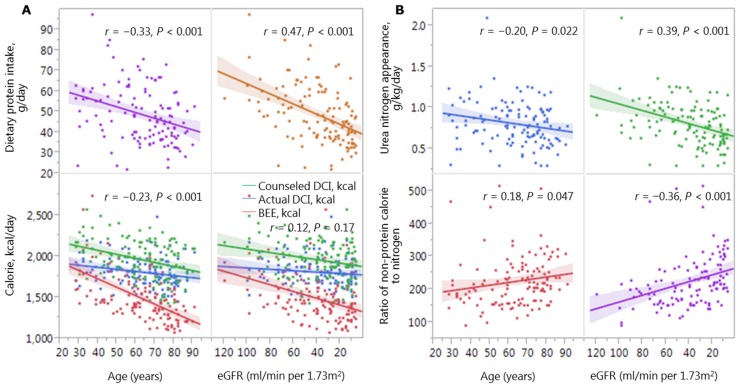
Age and GFR associated changes in (**A**) calorie and protein intake and (**B**) ratio of non-protein calories to nitrogen. Abbreviations: DCI, dietary calorie intake; BEE, basal energy expenditure; eGFR, estimated glomerular filtration rate.

Dietary protein intake was significant lower at older ages (*r* = −0.33, *p* < 0.001) and lower GFRs (*r* = 0.47, *p* < 0.001). In addition, calorie intake tended to be lower at older ages (*r* = −0.23, *p* < 0.05) (Figure 3A). Although the actual calorie intake was above the BEE, it remained below the counseled intake. Notably, the downward slopes in protein intake at older ages and lower GFRs were steeper than the slope in calorie intake. As a result, the NPC/N ratio tended to be higher at older ages (*r* = 0.18, *p* < 0.05) and lower GFRs (*r* = −0.36, *p* < 0.01) (Figure 3B).

### 3.4. Associations of GNRI with All-Cause Mortality, Cardiovascular Events, and Adverse Renal Outcomes

**Figure 4:**
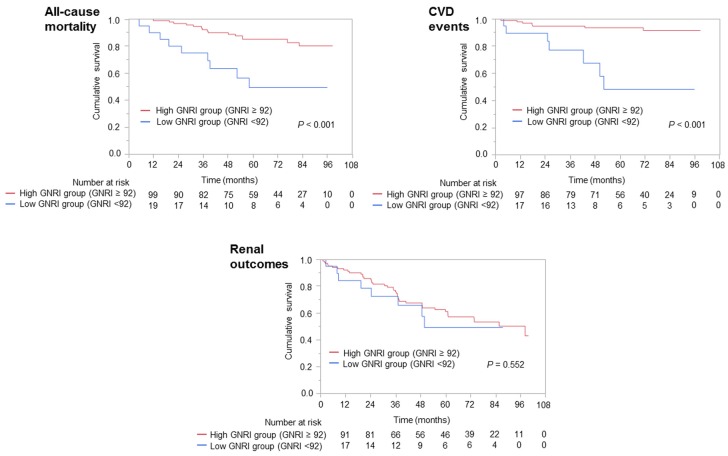
Kaplan–Meier analyses of all-cause mortality, cardiovascular events, and renal outcomes in the low and high GNRI groups. Abbreviations: CVD, cardiovascular disease; GNRI, geriatric nutritional risk index.

During a median 5.3-year follow-up period, 24 patients died, 14 experienced cardiovascular events, and 50 had adverse renal outcomes. Kaplan–Meier analysis showed significant differences in all-cause mortality and cardiovascular events, but not in renal outcomes, between the low and high GNRI groups (Figure 4). After adjustment for covariates, including age, diabetes mellitus, resistant hypertension, UPCR levels, NPC/N ratio, and baseline eGFR, GNRI < 92 was significantly associated with all-cause mortality (HR = 4.94; 95% CI = 1.61–15.42, *p* = 0.006) and cardiovascular events (HR = 9.37; 95% CI = 2.49–37.34, *p* < 0.001) (Table 3). Mortality rate was significantly higher in the low, than in the high, GNRI group (12.2 vs. 2.3 per 100 patient-years, *p* < 0.001). Seven patients (35%) in the low GNRI group (9.9 per 100 patient-years) and seven (6.6%) in the high GNRI group (1.2 per 100 patient-years) experienced cardiovascular events (*p* < 0.001). The rates of development of the composite renal end point were similar in the low (12.6 per 100 patient-years) and high (7.1 per 100 patient-years) GNRI groups (*p* = 0.55).

### 3.5. Independent Factors Associated with GNRI < 92

Univariate analysis showed that patient age, UPCR level, hyperuricemia, and the NPC/N ratio correlated with a GNRI < 92. Following multivariate analysis, UPCR and the NPC/N ratio remained independently associated with a GNRI < 92 (Table 4).

## 4. Discussion

The results of this study revealed that both dietary protein and calorie intake were significantly lower at older ages and lower GFRs in patients with CKD, and that the downward slope in protein intake was steeper than the slope in calorie intake, as we might induce a behavior modification for dietary intake balance between protein and energy. Consequently, the NPC/N ratio was higher at older ages and lower GFRs. Lower intake of dietary protein and calories were found to be significantly associated with lower GNRI, which, in turn, was significantly associated with body composition, all-cause mortality, and cardiovascular disease events, but not with adverse renal outcomes.

Clinical practice guideline for patients with CKD, defined as those with eGFR < 30 mL/min/1.73 m^2^, have recommended reducing protein intake to 0.8 g/kg/day in adults both with and without diabetes [1,2,30]. It reported that a protein-restricted diet supplemented with ketoanalogs of essential amino acids was effective and safe in ameliorating nitrogen waste product retention, as well as in delaying the renal replacement therapy initiation, with no deleterious effect on the nutritional status in selected patients with CKD [3,4]. In contrast, patients with advanced CKD, especially those below eGFR categories 4 and 5, frequently experience spontaneous reductions in dietary protein intake [31]. Frequent involuntary weight loss has been reported in outpatients over 65 years old, with a loss of >4% of body weight being an independent predictor of increased mortality [32]. These findings suggest the need for caution in restricting dietary protein in patients with advanced CKD. To date, however, no guidelines have been formulated regarding the discontinuation of dietary protein restrictions in these patients. This study found that the combination of an elevated NPC/N ratio and low dietary protein intake was associated with moderate or severe nutrition-related risk (GNRI < 92) even in patients receiving dietary counseling. Inadequate calorie intake was observed in 35.7% of these patients (Supplementary Materials Table S2), although most achieved BEE levels. Such patients with inadequate dietary intake were not always associated with adverse outcomes (data not shown). However, moderate or severe nutrition-related risk was linked to age-specific underweight and was associated with all-cause mortality and cardiovascular events. These findings suggest that dietary protein restriction may be inappropriate for patients with GNRI < 92 and that these patients should be counseled about dietary intake adequate to replenish protein and energy stores. Otherwise, any concomitant debilitating diseases and comorbidities in these malnourished patients may reduce life expectancy prognoses even if they retain the calorie intake.

Malnutrition and inflammation often coexist. Serum albumin concentration and BMI are often used as indicators of nutritional status in clinical practice [33,34,35]. However, both are influenced by several non-nutritional factors, including proteinuria, fluid status, and inflammation [36,37,38,39]. Therefore, measuring albumin or BMI alone may be insufficient to assess nutritional risk. In contrast, GNRI can minimize the multifactorial effects of each indicator by combining serum albumin and BMI. This study found that GNRI was associated with the NPC/N ratio and proteinuria, but not C-reactive protein. Low GNRI was associated with significantly higher rates of mortality and cardiovascular events, it was not associated with adverse renal outcomes, despite the inclusion of patients with massive proteinuria. These findings suggested that GNRI may be a useful predictor of patient prognosis, even in patients with CKD. Moreover, GNRI was positively correlated with water-free mass, consisting of proteins, fat, and minerals; ICW content; and ECW content. ICW content strongly depends on age and muscle mass, since the body cell mass is composed of a hydration component. With the decrease in water-free mass and ICW content, the percentage of the ECW content in the body weight relatively increased in the low GNRI group. Body composition in patients with CKD is characterized by excess ECW content associated with sodium retention and a decreased body cell mass with malnutrition, and we speculated that cell volume might be associated with nutritional status and indicate the reserve capacity for volume overload in patients with fluid volume imbalance [40,41]. In fact, the bioimpedance phase angle, as a measure of nutritional status [42,43], was associated with GNRI levels. Hyperphosphotemia is an independent risk factor for adverse outcomes in patients with CKD [44], and serum phosphorus level depends on protein intake [5]. In this study, the phosphorus level was strongly associated with kidney function rather than protein intake and nutritional status.

This study had several limitations. First, it was a retrospective cohort study conducted at a single center, and the sample size was small. However, detailed information was available on patients’ dietary records and body fluid composition and the follow-up period was relatively long. Second, GNRI has not been established as a consensus indicator of nutritional status in patients with CKD. However, our study found that GNRI was associated with body composition and was predictive of patient prognosis. GNRI was not compared with several other nutritional screening tools, such as Subjective Global Assessment and the Mini Nutritional Assessment Screening Form. These indices, however, entail subjective assessments, which may be affected by the examiner’s experience. In contrast, GNRI is an objective assessment, easily measured in patients with CKD. Third, we did not survey comorbidities, which might be associated nutritional status and adverse outcomes. Finally, the study design made it difficult to clearly differentiate between spontaneous reductions in dietary protein intake and restrictions of dietary protein. However, low dietary protein intake should be interpreted cautiously because of the inclusion in CKD populations of patients with inadequate dietary intake, especially older patients and advanced CKD.

## 5. Conclusions

This study showed that low dietary protein and calorie intake by subjects at older ages and with lower GFRs were associated with GNRI. GNRI was associated with body composition, all-cause mortality, and cardiovascular disease events, but not with adverse renal outcomes. Restricting protein intake may harm patients with low GNRI, and dietary prescription should be based on nutritional status and inflammatory activities. Improving nutritional status should be a priority in treating patients with any nutrition-related risks.

## Figures and Tables

**Table 1:** Baseline demographic and clinical characteristics in patients with GNRI ≥ 92 and < 92 at the time of dietary nutrient assessment.

Patient Characteristics	Low or No Nutrition-Related Risk (GNRI ≥ 92) *n* = 106 (84.1%)	Moderate or Severe Nutrition-Related Risk (GNRI < 92) *n* = 20 (15.9%)	*p*-Value
Age, years	62.1 ± 16.3	69.7 ± 14.3	0.043
Sex, male, *n* (%)	55 (51.9)	10 (50.0)	0.88
Time from first visit to the dietary clinic to the day of dietary recording, days	1829 ± 1691	1981 ± 1706	0.72
Height, cm	160 ± 10	156 ± 10	0.09
Weight, kg	60 ± 13	48 ± 7	<0.001
Body mass index, kg/m^2^	23.3 ± 3.8	19.6 ± 2.1	<0.001
Underling kidney disease			
Glomerulonephritis, *n* (%)	45 (42.5)	7 (35.0)	0.94
Diabetes mellitus, *n* (%)	14 (13.2)	4 (20.0)	
Nephrosclerosis, *n* (%)	22 (20.8)	4 (20.0)	
Others/Unknown, *n* (%)	25 (23.5)	5 (25.0)	
Systolic BP, mmHg	124 ± 16	123 ± 14	0.85
Diastolic BP, mmHg	72 ± 9	67 ± 11	0.10
Resistant high blood pressure, *n* (%)	23 (21.7)	5 (25.0)	0.74
Pulse pressure, mmHg	52 ± 12	56 ± 13	0.21
Blood urea nitrogen, mg/dL	29 ± 17	29 ± 17	0.87
Serum creatinine, mg/dL	1.97 ± 1.44	1.98 ± 1.34	0.99
eGFR_MDRD_, mL/min per 1.73 m^2^	40 ± 27	36 ± 24	0.48
GFR categories in KDIGO 2012			
G1 or 2, *n* (%)	26 (24.5)	3 (15.0)	0.56
G3a or G3b, *n* (%)	34 (32.1)	7 (35.0)	
G4, *n* (%)	23 (21.7)	7 (35.0)	
G5, *n* (%)	23 (21.7)	3 (15.0)	
Serum albumin, mg/dL	4.0 ± 0.4	3.3 ± 0.4	<0.001
Total cholesterol, mg/dL	193 ± 31	189 ± 56	0.76
Triglyceride, mg/dL	140 ± 84	120 ± 61	0.21
Fasting blood glucose, mg/dL	118 ± 36	148 ± 48	0.09
Uric acid > 7.0 mg/dL in males or >6.0 mg/dL in females, *n* (%)	60 (57.1)	16 (80.0)	0.06
Calcium, mg/dL	8.7 ± 0.6	8.7 ± 0.6	0.65
Phosphorus, mg/dL	3.4 ± 0.7	3.4 ± 0.6	0.65
C-reactive protein, mg/dL	0.2 ± 0.8	0.4 ± 0.6	0.43
Hemoglobin, g/dL	12.3 ± 2.0	11.1 ± 1.5	0.006
UPCR, g/g Cr	0.8 ± 1.3	1.8 ± 2.1	0.045
Urea nitrogen appearance, g/kg per day	0.84 ± 0.20	0.89 ± 0.31	0.53

Abbreviations: GNRI, geriatric nutritional risk index; BP, blood pressure; eGFR_MDRD_, estimated glomerular filtration rate by the Modification of Diet in Renal Disease method; KDIGO, KIDNEY DISEASE|IMPROVING GLOBAL OUTCOMES; UPCR, urinary protein-to-creatinine ratio.

**Table 2:** Baseline dietary intake in patients with GNRI ≥ 92 and < 92 at the time of dietary nutrient assessment.

Patients Characteristics	No Nutrition-Related Risk (GNRI ≥ 92) *n* = 106 (84.1%)	Nutrition-Related Risk (GNRI < 92) *n* = 20 (15.9%)	*p*-Value
Sodium intake, mg/day	3077 ± 1,210	2942 ± 1,139	0.65
Sodium intake per BW, mg/kg/day	52 ± 19	64 ± 26	0.07
Protein intake, g/day	47 ± 15	41 ± 15	0.09
Protein intake per BW, g/kg/day	0.80 ± 0.24	0.87 ± 0.35	0.37
Protein intake per ideal BW g/standard weight/day	0.83 ± 0.26	0.77 ± 0.29	0.36
Calorie intake, kcal/day	1819 ± 189	1763 ± 129	0.11
Calorie intake per BW, kcal/kg/day	31 ± 5	38 ± 4	<0.001
Calorie intake per ideal BW, kcal/standard weight/day	32 ± 3	33 ± 3	0.19
Ratio of non-protein calories to nitrogen	218 ± 71	261 ± 86	0.043
Basal energy expenditure, kcal	1530 ± 285	1270 ± 140	<0.001

Abbreviations: GNRI, geriatric nutritional risk index; BW, body weight.

**Table 3:** Hazard risks of GNRI < 92 for all-cause mortality and cardiovascular events.

Variables	HR (95% CI)	*p*-Value
All-cause death		
Unadjusted	3.87 (1.62–8.72)	0.003
Age-adjusted	3.91 (1.62–8.94)	0.003
Multivariable-adjusted ^a^	5.95 (1.94–18.04)	0.002
Cardiovascular disease event		
Unadjusted	8.26 (2.73–25.84)	<0.001
Age-adjusted	13.44 (3.99–50.51)	0.001
Multivariable-adjusted ^b^	9.48 (2.47–44.31)	<0.001

Abbreviations: GNRI, geriatric nutritional risk index; HR, hazard ratio; CI, confidence interval. ^a^ Adjusted for age, diabetes mellitus, C-reactive protein, urinary protein-to-creatinine ratio, ratio of non-protein calorie to nitrogen, hemoglobin concentration, and baseline estimated glomerular filtration rate (A graphic approach (log–log plots) was used to assess the proportionality of HRs. Continuous variables were divided into two groups by their median values. All variables in which HR was estimated satisfied this assumption. If serum albumin concentration or phosphorus levels were used as the dependent variable, results remained similar (data not shown)); ^b^ Adjusted for age, diabetes mellitus, resistant high blood pressure, C-reactive protein, urinary protein-to-creatinine ratio, ratio of non-protein calorie to nitrogen, hemoglobin concentrations, and baseline estimated glomerular filtration rate (A graphic approach (log–log plots) were used to assess the proportionality of HRs. Continuous variables were divided into two groups by their median values. All variables in which HR was estimated satisfied this assumption. If serum albumin concentration or phosphorus levels were used as the dependent variable, results remained similar (data not shown)). The time from first visit to the dietary clinic to the day of dietary recording was not associated with the investigated outcomes on univariate analyses. If this interval was used as the dependent variable, the results remained similar (data not shown).

**Table 4:** Factors independently associated with GNRI < 92.

Variables	Univariate Analysis		Multivariate Analysis	
	OR (95% CI)	*p*-Value	OR (95% CI)	*p*-Value
Age, per 10 years of age	0.77 (0.54–1.05)	0.12	0.85 (0.57–1.20)	0.36
UPCR, g/g Cr	0.69 (0.51–0.92)	0.01	0.71 (0.52–0.96)	0.03
Uric acid > 7.0 mg/dL in males or >6.0 mg/dL in females	2.89 (0.98–10.60)	0.05	2.10 (0.66–8.12)	0.21
Ratio of non-protein calorie to nitrogen, per 10 units	0.94 (0.88–0.99)	0.02	0.93 (0.87–0.99)	0.04

Abbreviations: GNRI, geriatric nutritional risk index; OR, odds ratio; CI, confidence interval; UPCR, urinary protein-to-creatinine ratio.

## Supplementary Materials

The following are available online at http://www.mdpi.com/2072-6643/8/10/662/s1, Table S1: Body fluid composition in patients with GNRI ≥ 92 and < 92 at the time of dietary nutrient assessment, Table S2: Demographic and clinical characteristics of patients who did and did not achieve recommended daily protein intake.

## Abbreviations

The following abbreviations are used in this manuscript:

BEE	basal energy expenditure
BIA	bioimpedance analysis
BMI	body mass index
CKD	chronic kidney disease
CI	confidence interval
Cr	serum creatinine
ECW	extracellular water
eGFR	estimated glomerular filtration rate
GNRI	geriatric nutritional risk index
HR	hazard ratio
ICW	intracellular water
MDRD	Modification of Diet in Renal Disease
NPC/N	non-protein calorie to nitrogen
PEW	protein-energy wasting
TBW	total body water
UNA	urea nitrogen appearance
UPCR	urinary protein-to-creatinine ratio
