# Supplementary material for: Association between Low Dietary Protein Intake and Geriatric Nutrition Risk Index in Patients with Chronic Kidney Disease: A Retrospective Single-Center Cohort Study
Source: Nutrients. 2016 Oct 23;8(10):662. doi: 10.3390/nu8100662 (PMC5084048; doi:10.3390/nu8100662)
Supplement: Supplementary file 1 [file nutrients-08-00662-s001.docx]

Supplementary Materials: Association between Low Dietary Protein Intake and Geriatric Nutrition Risk Index in Patients with Chronic Kidney Disease:
A Retrospective Single-Center Cohort Study

Aki Kiuchi, Yasushi Ohashi, Reibin Tai, Toshiyuki Aoki, Sonoo Mizuiri, Toyoko Ogura,
Atsushi Aikawa and Ken Sakai

**Table S1.** Body fluid composition in patients with a geriatric nutritional risk index (GNRI) ≥ 92 and
< 92 at the time of dietary nutrient assessment.

| **Patient Characteristics** | **Low or No Nutrition-Related Risk (GNRI ≥ 92) *n* = 106 (84.1%)** | **Moderate or Severe Nutrition-Related Risk (GNRI < 92) *n* = 20 (15.9%)** | ***p*-Value** |
| --- | --- | --- | --- |
| Phase angle, ° | 5.1 ± 0.9 | 4.4 ± 0.6 | <0.001 |
| Total Body Water, L | 31.7 ± 6.9 | 27.6 ± 4.2 | <0.001 |
| % in body weight | 53.3 ± 6.4 | 58.0 ± 4.5 | <0.001 |
| Intracellular water, L | 19.3 ± 4.3 | 16.5 ± 2.5 | <0.001 |
| % in body weight | 32.3 ± 4.1 | 34.7 ± 2.8 | 0.003 |
| Extracellular water, L | 12.4± 2.7 | 11.1 ± 1.7 | 0.006 |
| % in body weight | 20.9 ± 2.5 | 23.3 ± 1.9 | <0.001 |
| Water-free mass, kg | 28.1 ± 7.9 | 20.0 ± 4.2 | <0.001 |
| % in body weight | 46.8 ± 6.4 | 42.0 ± 4.5 | <0.001 |
| Ratio of extracellular water to intracellular water | 0.65 ± 0.03 | 0.67 ± 0.02 | <0.001 |

Abbreviations: GNRI, geriatric nutritional risk index.

**Table S2.** Demographic and clinical characteristics of patients who did and did not achieve recommended daily protein intake.

| **Patients’ Characteristics** | **DPI < 0.6 g/kg/Day or DPI 0.6–0.8 g/kg/Day and DCI < 30–35 kcal/Standard Weight/Day *n* = 45 (35.7%)** | **DPI 0.6–0.8 g/kg/Day and DCI 30–35 kcal/Standard Weight/Day *n* = 38 (30.0%)** | **DPI > 0.8 g/kg/Day *n* = 45 (35.7%)** | ***p*-Value** |
| --- | --- | --- | --- | --- |
| Age, years | 65.5 ± 16.3 | 64.7 ± 16.5 | 60.1 ± 15.8 | 0.25 |
| Sex, male, *n* (%) | 29 (64.4) | 18 (50.0) | 18 (40.0) | 0.07 |
| Diabetes mellitus, *n* (%) | 9 (20.0) | 6 (16.7) | 3 (6.7) | 0.17 |
| Body mass index, kg/m^2^ | 22.7 ± 4.5 | 22.9 ± 3.0 | 21.5 ± 3.4 | 0.022 |
| Systolic BP, mmHg | 127 ± 16 | 123 ± 18 | 121 ± 14 | 0.20 |
| Diastolic BP, mmHg | 73 ± 11 | 70 ± 9 | 70 ± 8 | 0.25 |
| Blood urea nitrogen, mg/dL | 31 ± 16 | 30 ± 19 | 25 ± 15 | 0.87 |
| Serum creatinine, mg/dL | 2.51 ± 1.59 | 1.97 ± 1.41 | 1.44 ± 1.03 | 0.001 |
| eGFR_MDRD_, ml/min per 1.73 m^2^ | 31 ± 23 | 38 ± 26 | 50 ± 27 | 0.002 |
| Serum albumin, mg/dL | 3.9 ± 0.4 | 3.9 ± 0.5 | 4.0 ± 0.5 | 0.26 |
| Total cholesterol, mg/dL | 191 ± 34 | 194 ± 39 | 191 ± 37 | 0.95 |
| Triglyceride, mg/dL | 152 ± 99 | 126 ± 60 | 131 ± 73 | 0.29 |
| Fasting blood glucose, mg/dL | 122 ± 38 | 122 ± 34 | 128 ± 51 | 0.89 |
| Hemoglobin, g/dL | 11.7 ± 1.9 | 12.1 ± 2.3 | 12.5 ± 1.6 | 0.19 |
| UPCR, g/g·Cr | 1.2 ± 1.3 | 1.1 ± 1.7 | 0.7 ± 1.5 | 0.35 |
| Geriatric nutritional risk index | 102 ± 11 | 101 ± 10 | 101 ± 10 | 0.74 |

Abbreviations: DPI, dietary protein intake; DCI, dietary calorie intake; BP, blood pressure; eGFR_MDRD_, estimated glomerular filtration rate by the modification of diet in renal disease method; UPCR, urinary protein-to-creatinine ratio.
